# Research Progress on the Influence of Surface Treatment Techniques on Fatigue Properties of Titanium Alloys

**DOI:** 10.3390/ma19081511

**Published:** 2026-04-09

**Authors:** Baicheng Liu, Hongliang Zhang, Xugang Wang, Yubao Li, Shenghan Li, Xue Cui, Yurii Luhovskyi, Zhisheng Nong

**Affiliations:** School of Materials Science and Engineering, Shenyang Aerospace University, Shenyang 110136, China; 15942786975@126.com (B.L.); 13070717117@163.com (X.W.); 18841204378@163.com (Y.L.); m15541529917@163.com (S.L.); papercx@163.com (X.C.); luhovskyi@gmail.com (Y.L.)

**Keywords:** titanium alloy, fatigue performance, coating preparation, surface strengthening

## Abstract

Titanium alloys exhibit exceptional strength-to-density ratios, high hardness, and outstanding resistance to elevated temperatures, making them indispensable structural materials in aerospace engineering, marine construction, and biomedical applications. In aerospace systems specifically, fatigue failure represents the predominant failure mode for titanium alloy components. This review systematically examines prevalent surface treatment techniques for titanium alloys—including shot peening, ultrasonic rolling treatment, hot isostatic pressing (HIP), physical vapor deposition (PVD), micro-arc oxidation (MAO), and thermal spray processes—and critically evaluates their respective effects on fatigue performance. The underlying mechanisms of each technique are concisely outlined, with emphasis on stress state evolution, near-surface microstructural refinement, and interfacial integrity. Building upon the characteristic surface-dominated fatigue fracture behavior of titanium alloys, this work focuses on how coating composition, architecture (e.g., graded, multilayer, or nanocomposite designs), and interfacial bonding strength govern fatigue resistance. A unified analysis is presented on the distinct yet complementary roles of substrate deformation strengthening (e.g., residual compression, grain refinement) and coating-mediated protection (e.g., barrier function, crack deflection, stress redistribution) during fatigue crack initiation and propagation. Key determinants of fatigue performance, including residual stress distribution, coating/substrate adhesion, thermal mismatch, and environmental degradation susceptibility, are rigorously assessed. Finally, emerging research frontiers are identified, including intelligent process–structure–property mapping, in situ monitoring of fatigue damage at coated interfaces, and design of multifunctional gradient coatings that synergistically enhance strength, wear resistance, and fatigue endurance of titanium alloy components.

## 1. Introduction

In recent years, titanium alloys have been widely utilized in various fields, including aerospace, marine engineering, chemical equipment, and the biomedical industry, owing to their remarkable specific strength, superior fatigue resistance, and excellent toughness [1,2,3]. As the aerospace, transportation, and defense industries enter a phase of rapid development, various advanced equipment has placed more stringent requirements on adaptability to service environments, especially under extreme operating conditions such as high temperature (>650 °C) and high pressure (>20 MPa) [4]. Titanium alloy components and workpieces must exhibit long-term structural stability, functional reliability, and exceptional service durability—requirements that are steadily intensifying across aerospace, biomedical, and energy sectors [5]. This trend has driven increasingly stringent requirements for the comprehensive performance of titanium alloys, requirements that encompass not only fundamental mechanical properties but also robust functionality under extreme environmental conditions and in complex operational scenarios [6,7,8,9]. However, titanium alloys suffer from intrinsic performance limitations, including low surface hardness, insufficient wear resistance, and a pronounced degradation in fatigue strength at elevated temperatures relative to room temperature [10]. These limitations are further exacerbated during service under extreme conditions involving friction and impact—leading readily to surface wear, micro-scratching, or even catastrophic component failure. Such degradation not only markedly reduces equipment service life but also jeopardizes operational safety and system-level stability, constituting a critical bottleneck to the broader deployment of titanium alloys in extreme-environment applications [11].

The primary failure modes of titanium alloys encompass fatigue failure, fretting wear failure, high-temperature fatigue failure, and corrosion fatigue failure, among others [12,13,14]. Among these, fatigue failure stands as one of the dominant failure modes of titanium alloys. Fatigue failure of titanium alloys is a core factor inducing mechanical malfunctions and driving material property degradation—it not only severely impairs the operational reliability and stability of working components but also drastically shortens their actual service life, posing a potential threat to the overall operational efficiency and safety of equipment [15].

Particularly under extreme service conditions—including elevated temperature, high pressure, cyclic mechanical loading, and corrosive environments—the synergistic degradation effects markedly accelerate fatigue failure. Specifically, such conditions accelerate surface damage accumulation (e.g., oxidative wear and plastic deformation), thereby degrading near-surface integrity, and promote preferential microcrack nucleation at the material surface. Under sustained cyclic stress, these surface-initiated microcracks propagate, coalesce, and ultimately evolve into macroscopic fatigue cracks, leading to a substantial reduction in fatigue life [16]. Ultimately, this process triggers rapid fatigue failure of the material, and may even lead to sudden damage of key components, inducing severe mechanical failures [17].

Consequently, substantial scholarly attention has been devoted to investigating the fatigue behavior and underlying mechanisms of titanium alloys, leading to the identification of several empirically validated patterns. Given the critical requirement for reliable and sustained performance of structural components, surface strengthening of titanium alloys constitutes an essential engineering strategy [18,19,20,21]. Titanium alloys are routinely subjected to surface strengthening treatments or protective coating deposition to extend their service life, owing to intrinsic material limitations, namely, low inherent hardness, poor tribological performance, and limited resistance to high-temperature oxidation [22]. The fatigue failure mechanism in titanium alloys proceeds through a well-defined sequence: accumulation of surface damage, initiation of microcracks, propagation of cracks, and eventual fracture [23]. Surface deformation strengthening induces plastic deformation in the material’s surface layer, introduces residual compressive stress, and modifies both surface morphology and subsurface microstructural states [24], thereby enabling the regulation and enhancement of the material’s fatigue performance [25].

The regulatory effect of coatings on the fatigue performance of titanium alloys is not determined by a single factor but arises from the synergistic interplay among multiple interdependent parameters—namely, intrinsic coating properties, fabrication processes, interface architecture, and in-service environmental conditions. Among these, coating adhesion strength, fracture toughness, interfacial compatibility, and process-induced microstructural features constitute the primary determinants of fatigue behavior [26,27,28]. Crucially, coatings function as protective barriers that impede direct exposure of the titanium alloy substrate to deleterious external stimuli, including mechanical wear and friction, corrosive media, and high-temperature oxidative environments. By suppressing the formation of fatigue-critical surface defects such as wear-induced scratches, corrosion-driven pitting, and oxidation-induced embrittlement layers, coatings effectively eliminate or reduce key crack nucleation sites, thereby mitigating fatigue failure at its inception [29].

Currently, common surface deformation strengthening techniques include conventional shot peening [30,31,32,33], laser shock peening [34,35,36,37,38], and ultrasonic rolling [39,40]. Experimental findings [41,42,43,44,45] indicate that these techniques essentially induce irreversible plastic flow in the surface metal layer via external force application, thereby forming a residual compressive stress field, refining grain structures, optimizing surface quality, and reducing the initiation of surface microcracks—collectively leading to a significant enhancement in the material’s fatigue resistance [39,46,47,48].

Common coating preparation technologies at present include physical vapor deposition (PVD) [26,27,49,50], laser cladding [51,52,53], and micro-arc oxidation (MAO) [54,55,56,57,58]. At present, systematic research on the influence of surface treatment processes on the fatigue performance of titanium alloys is still relatively scarce. This review focuses on two mainstream surface modification methods: surface deformation strengthening technology and coating treatment, systematically summarizing their effects on the fatigue strength, fatigue life, and high-temperature service stability of titanium alloys [59,60,61]. The aim is to provide theoretical support and technical references for the development of titanium alloy materials with both high fatigue strength and excellent high-temperature stability.

## 2. Methods

**Figure 1:**
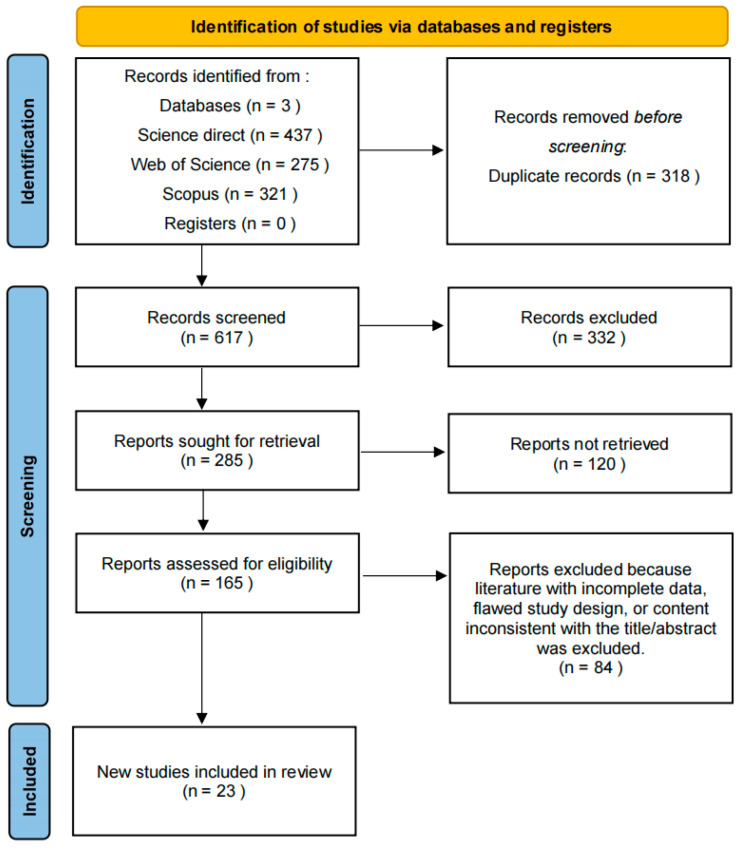
Flowchart of the literature retrieval and screening process.

The literature search and screening procedures of this review strictly adhere to the standardized framework for systematic reviews, thereby ensuring the comprehensiveness, accuracy, and reliability of data acquisition. Meanwhile, the flowchart formatted in accordance with the PRISMA guidelines is presented in Figure 1. The specific steps are outlined as follows:

Literature Search Strategy: The search scope was restricted to three core academic databases, namely ScienceDirect, Web of Science, and Scopus. The search keywords were defined as “titanium alloy”, “surface peening”, “micro-arc oxidation”, “additive manufacturing”, and “fatigue performance”. A combined keyword retrieval approach was employed to acquire relevant literature, with the publication period limited to the past decade—this was intended to maximize the inclusion of published research findings in the field.

Literature Screening Procedure: A two-step screening approach was employed to identify the final included literature. The first step is preliminary screening, where titles and abstracts undergo rapid review: documents explicitly mentioning non-titanium alloy materials, non-surface strengthening systems, or not focusing on fatigue performance optimization are directly excluded. If the abstract indicates non-experimental or non-review studies (e.g., theoretical hypotheses, conference abstracts), or deviates from the theme of “fatigue performance of titanium alloys” in terms of research subjects and methods, or omits key fatigue performance data (e.g., fatigue strength, fatigue life), they are preliminarily excluded. Papers meeting the inclusion criteria in titles and abstracts proceed to the second screening stage. The second step is secondary screening. After obtaining full-text articles that passed the initial screening, researchers conduct rigorous verification of core content (including research background, research subjects, materials and methods, experimental data, and conclusions) to ensure compliance with inclusion criteria: confirming whether the research subjects are titanium alloys and whether the focus is on the impact of surface strengthening on fatigue performance; verifying whether the research methods adhere to academic norms and whether complete experimental procedures (e.g., surface treatment parameters, fatigue performance testing protocols) and raw data are provided; excluding articles with incomplete data (e.g., missing key experimental conditions or performance indicators), design flaws (e.g., lack of control groups, insufficient repeated experiments), or discrepancies between actual research content and titles/abstracts (e.g., theme deviation, data incongruence with conclusions). Papers fully meeting the inclusion criteria are labeled as “included articles” and proceed to the subsequent data extraction phase.

Data Extraction and Validation: A standardized, protocol-guided framework was adopted to systematically extract critical information from the included studies. The extraction schema follows a rigorously defined sequence—“preparation method → material composition → experimental conditions → performance metrics → research conclusions”—and comprehensively encompasses fatigue-specific parameters: test configuration (e.g., counterbody material, applied load, stress ratio R, environmental medium), quantitative fatigue outcomes (e.g., fatigue life N_f_, fatigue strength σ_f_ at a specified life criterion), and principal mechanistic interpretations. Dual-layer validation was rigorously conducted to ensure data integrity: First, intra-study consistency was verified by cross-checking reported results under identical or nominally equivalent experimental conditions (e.g., agreement among fatigue life values measured at the same nominal stress amplitude across replicate specimens); second, inter-study coherence was assessed by comparing fatigue performance data generated under comparable testing protocols and material systems, with observed discrepancies systematically attributed to methodological variables—including measurement uncertainty of fatigue testing equipment, ambient temperature and humidity control, fidelity of stress ratio application, specimen surface condition and dimensional tolerances, and prior thermomechanical history. Any identified inconsistencies prompted immediate re-examination of the original source publication to correct extraction errors, thereby guaranteeing traceability, reproducibility, and analytical fidelity of the final dataset.

Literature Evaluation and Classification: Based on the extracted standardized data, the included literature was categorized and organized by preparation method, covering mainstream processes such as shot peening (SP), ultrasonic surface rolling treatment (USRT), physical vapor deposition (PVD), micro-arc oxidation (MAO), and additive manufacturing (AM). The research progress regarding the effects of each surface treatment method on the fatigue performance of titanium alloys was summarized separately. This literature review meticulously adheres to the PRISMA checklist requirements for all cited references, thereby ensuring the rigor and reliability of the synthesis (Supplementary Materials).

Fatigue strength is the critical threshold of bearing capacity that a material needs to resist fatigue failure. Under the specified constant amplitude cyclic loading conditions, with the agreed failure criterion and the designated cyclic life base, the specimen can withstand the maximum cyclic stress amplitude without experiencing the specified fatigue failure. Fatigue life is the durability index of a material against fatigue failure under the given service load. It is the total number of complete cycle loads experienced by the specimen from the start of cyclic loading until the specified failure criterion is reached, and it is the cycle count-based indicator of the material’s fatigue durability limit. Both are indispensable for reflecting the fatigue performance of the material.

## 3. Surface Strengthening Techniques

**Figure 2:**
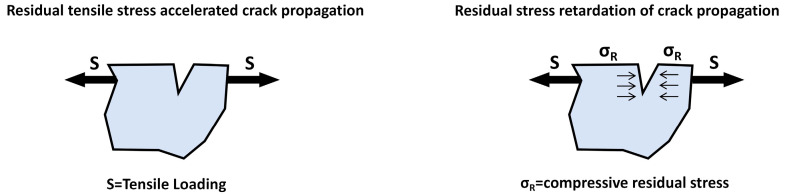
Effect of residual stress on crack initiation mechanism and mechanical strengthening on material roughness and fatigue performance.

In the study of fatigue properties of titanium alloys, fatigue strength and fatigue life are the primary research subjects. The superiority or inferiority of the fatigue properties of titanium alloys is primarily reflected in two critical indices: fatigue strength (σ_s_) and fatigue life (N_f_) [62]. Table 1 summarizes the detailed information on the effects of different surface strengthening techniques on the fatigue life (cycle number) of titanium alloys under the same stress amplitude. The surface roughness significantly affects the fatigue performance of titanium alloys. Higher roughness values lead to shorter fatigue life. However, surface strengthening treatments not only increase surface roughness but also reduce residual stresses and refine grain structure, thereby enhancing both fatigue strength and fatigue life. Figure 2 elucidates the impact of residual stress on the crack initiation mechanism as well as the role of mechanical strengthening in influencing material surface roughness and fatigue performance. In comparison to other contributing factors, residual stress plays a predominant role in governing crack initiation.

Table 2 summarizes detailed information regarding the effects of different surface strengthening techniques on the fatigue strength of titanium alloys under the same cycle number.

### 3.1. Effect of Shot Peening on Fatigue Performance of Titanium Alloys

Shot peening (SP), also referred to as shot peening strengthening, is an effective technique for mitigating fatigue and extending the service life of components [63,85,95]. By precisely controlling the shot material, impingement pressure, and velocity, the plastic deformation induced on the material surface significantly elevates the dislocation density in the surface layer, thereby increasing surface hardness and yield strength, enhancing the surface layer’s resistance to plastic deformation, and reducing local stress concentration under fatigue loading. The introduced residual compressive stress can counteract a portion of the tensile stress from external loads, lowering the driving force for surface crack initiation; furthermore, even if cracks initiate, residual compressive stress inhibits their further propagation, thus substantially improving the material’s fatigue life. However, excessively high impingement pressure or oversized shots may lead to excessive plastic deformation in the titanium alloy surface layer, or even generate new microcracks. Hence, selecting an appropriate shot peening regime for different titanium alloy materials is of critical importance.

Currently, the mainstream shot peening techniques include conventional shot peening (CSP), laser shock peening (LSP), and ultrasonic shock peening (USP). In conventional shot peening, mechanical kinetic energy drives the process, enabling a fatigue life improvement of approximately 50–200% for titanium alloys. In contrast, laser shock peening and ultrasonic shot peening utilize high-power pulsed lasers and ultrasonic vibration systems, respectively, to achieve a more significant enhancement in the fatigue life of titanium alloys.

#### 3.1.1. Shot Peening (SP) Treatment

**Figure 3:**
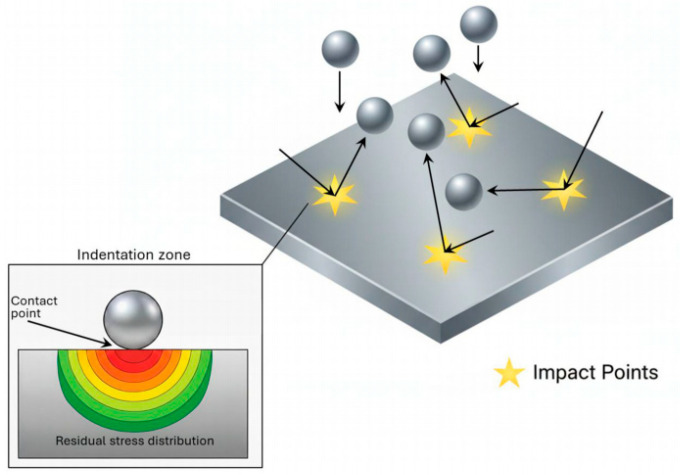
Schematic diagram of shot peening (SP) processing [96].

In the surface modification study of Ti6Al4V alloy, Qi et al. [63] employed unilateral and bilateral shot peening (SP) treatments. Figure 3 illustrates the SP processing method.

Shot peening is a surface strengthening technique that bombards the material surface with high-velocity hard particles. Repeated impacts induce numerous micro-dimples and irregular undulations on the surface, which directly increase the arithmetic mean deviation of the surface profile and thus raise the surface roughness Ra. The roughened surface features more micro-peaks and micro-valleys. When brought into contact with another surface, these micro-peaks provide a greater number of actual contact points compared with a smooth surface. Since the external load is distributed over a larger number of contact points, the load carried by each individual contact point is reduced. Fatigue cracks in the as-received components predominantly originated from stress concentration regions, with multiple cracks present. After SP treatment, cracks in the specimens only initiated at the edges of the contact zone, and no wear pits were observed on the surface, thus improving the fatigue life of the specimens.

Wang et al. [65] performed shot peening on the surface of titanium alloy rods. Post-treatment characterization revealed that the surface roughness Ra increased to 1.29 μm, with a surface residual compressive stress (σ_crs_) of −450 MPa. However, the SP process induced numerous surface pits, which triggered localized stress concentration. The detrimental effects arising from these pits outweighed the beneficial contributions of residual compressive stress and grain refinement, leading to elevated fatigue risk, non-uniform plastic deformation, and failure to form an effective, homogeneous strengthening layer. Consequently, the fatigue life of the treated rods was slightly lower than that of the base material. Evidently, optimization of the SP regime is necessary to mitigate surface defects and prevent stress concentration from counteracting the strengthening effects.

Ji et al. [81] applied shot peening to the surface of a titanium alloy. Post-treatment analysis showed that the surface roughness Ra increased to 1.286 μm, while the surface grain size in the microstructure increased and a grain refinement layer was formed. This observation indicated an enhancement in the fatigue performance of the SP-treated titanium alloy: its fatigue life was extended from σ_a_ = 520 MPa to σ_a_ = 610 MPa, corresponding to an approximate 17% improvement in fatigue strength. Under high stress, cracks initiated from surface pits; under low stress, however, the initiation site shifted to the subsurface region.

#### 3.1.2. Laser Shock Peening (LSP) Treatment

**Figure 4:**
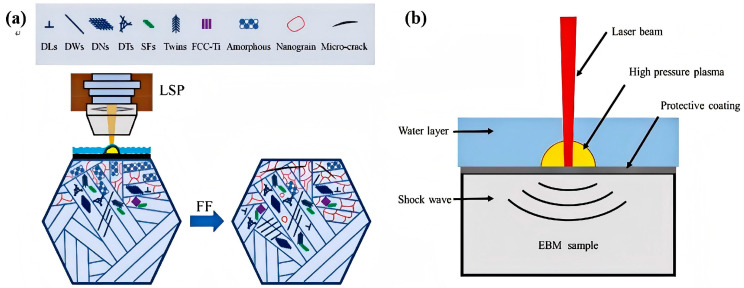
Schematic illustrations of laser shock peening (LSP) treatment: LSP treatment applied to the titanium alloy surface (**a**) [73], water-assisted LSP treatment (**b**) [34].

Laser shock peening emerges as a pivotal technology for enhancing the fatigue life of titanium alloys. This advantage stems from its inherent characteristics: a low risk of inducing plastic deformation in thin-walled components, uniform stress distribution across the treated specimen surface, formation of nanocrystalline structures in the surface layer, and a pronounced fine-grain strengthening effect that effectively inhibits crack propagation. Figure 4 presents a schematic illustration of the LSP process: a laser beam irradiates the confinement layer on the workpiece surface, causing the absorption layer to undergo instantaneous vaporization and form high-temperature, high-pressure plasma. The expansion of this plasma generates intense shock waves that transfer energy to the workpiece [34,36,73,85].

In the research on surface modification of Ti6Al4V alloy, Zhang et al. [85] performed LSP on the surface of annealed titanium alloy and compared the microhardness under untreated, single-impact, and double-impact conditions. The microhardness was enhanced by 14% after a single impact and 24% after double impacts. The results revealed that the fatigue life of the titanium alloy subjected to single-impact treatment increased by 22.2% compared to the untreated counterpart, while that treated with double impacts exhibited a 41.7% improvement.

Feng et al. [86] carried out LSP and Waterless Laser Shock Peening (WLSP) on the titanium alloy surface. Traditional LSP employs water as the confinement layer, which is susceptible to vaporization and failure at elevated temperatures. In contrast, WLSP utilizes a 2 mm thick BK7 glass as the confinement layer, coupled with a thick aluminum absorption layer for surface protection. illustrates the formation of a nanocrystalline layer on the WLSP-treated surface, along with an increased dislocation density. Consequently, the high-cycle vibration fatigue strength was elevated from σ_a_ = 399 MPa to σ_a_ = 568 MPa.

The research team led by Altenberger [83] explored the influence of LSP treatment on the high-temperature fatigue performance of titanium alloy. At a test temperature of 550 °C, comparative experiments between Deep Rolling (DR) and LSP treatments were conducted. The fatigue strength of DR treatment at 550 °C was comparable to that of LSP; however, the twin-free dislocation structure induced by LSP demonstrated superior resistance to high-temperature creep compared to the deformed twin structure of DR. The titanium alloy treated solely with LSP achieved the highest high-temperature fatigue strength. Hu et al. [36] predicted the fatigue performance of LSP-treated titanium alloy via a model, further quantifying various strengthening mechanisms to provide guidance for the optimization of LSP processes and maximize fatigue life enhancement.

In the modification research of Ti11 alloy, Nie and Luo et al. [87,88] applied LSP technology to achieve surface deformation strengthening of Ti11 alloy. The study by Nie et al. reveals distinct regions in untreated fracture surfaces: the crack initiation zone, fatigue crack propagation zone, and final fracture zone, with cracks originating from the specimen surface. In contrast, the corresponding regions in laser shock peening (LSP)-treated specimens exhibit sub-surface crack initiation, which contributes to enhanced fatigue life. Additionally, LSP treatment leads to reduced fatigue crack spacing and promotes the formation of secondary cracks. These secondary cracks impede the propagation of the primary crack, thereby improving fatigue strength. Following laser shock processing, the fatigue crack spacing becomes narrower, and the fatigue crack path exhibits greater tortuosity, resulting in increased fatigue resistance.

#### 3.1.3. Ultrasonic Shock Peening (USP) Treatment

Ultrasonic Shock Peening (USP) is primarily applied in the fields of titanium alloy thin-walled components and thin-walled blades. This is ascribed to its prominent advantages, including low-energy high-frequency impact, gentle plastic deformation of the surface layer, and a reduced risk of deformation for thin-walled parts [95].

The fundamental principle of USP involves the utilization of an ultrasonic vibration system: micro-shot pellets transmit energy via high-frequency impacts driven by the vibration rod. Under the action of such energy impacts, the surface metal accumulates plastic deformation, leading to a more uniform stress distribution.

**Figure 5:**
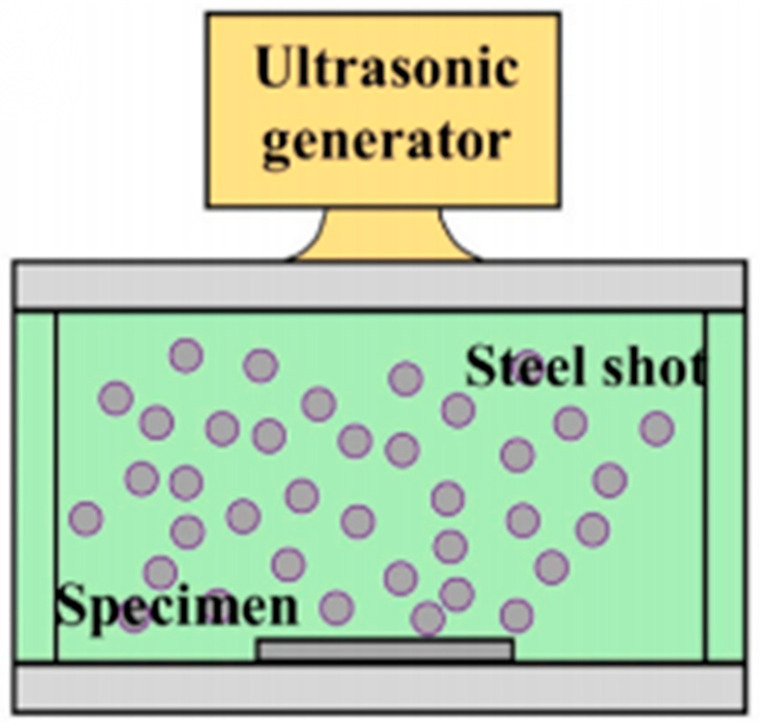
Schematic illustration of ultrasonic shock peening (USP) treatment [97].

Figure 5 presents a schematic diagram of the USP process. After treatment, an ultrafine-grained layer is formed on the surface, with a uniform dislocation distribution, while strong toughness is retained.

Wu et al. [75] applied Ultrasonic Shock Peening (USP) to TA15 alloy fabricated via laser melting deposition. Post-treatment, the TA15 alloy exhibited the formation of high-density dislocations and deformation twins, accompanied by enhancements in surface hardness and fatigue life. Under identical stress magnitudes, the fatigue life increased by 200% and 43%, respectively. This observation indicates that USP treatment reduces the crack propagation rate of TA15 alloy, thereby improving its fatigue performance. Kumar et al. [74] conducted USP treatment on TC4 alloy, which resulted in grain refinement and increased surface roughness.

### 3.2. Effect of Ultrasonic Rolling Treatment on the Fatigue Properties of Titanium Alloys

Ni’s research team [40] investigated TC4 alloy with an MAO coating subjected to ultrasonic surface rolling processing (USRP). Post-treatment, the void distribution within the MAO coating was homogenized; notably, the fatigue life was significantly enhanced under low-stress conditions, while under high-stress conditions, the fatigue life of the substrate was comparable to that of the treated specimens. presents the fatigue life of MAO-coated titanium alloy versus that of USRP-treated counterparts, indicating that the MAO coating only modulates the surface stress of the material, whereas USRP treatment stabilizes the deep-seated stress of the material.

Ao et al. [90] explored the effect of USRP treatment cycles on the fatigue performance of titanium alloys. The surface roughness (Ra = 0.5 μm) of titanium alloys treated with 1 cycle of USRP was comparable to that of those treated with 2 cycles, and both exhibited higher surface hardness than the substrate. reveals that under identical stress levels, the fatigue life of USRP-treated titanium alloys exceeded that of the substrate, suggesting that USRP treatment reduces the crack propagation rate and induces a slow propagation zone.

Liu et al. [91] investigated the influence of USRP on the fatigue performance of titanium alloys. Post-treatment, the surface roughness increased by 72.6%, microhardness was significantly improved, and a gradient plastic deformation layer was formed on the surface. demonstrates that the fatigue limit of USRP-treated titanium alloys at 10^7^ cycles increased from 500 MPa (substrate) to 610 MPa, and at a stress of 700 MPa, the fatigue life reached 7.6 × 10^5^ cycles.

Liu’s research team [92] also examined the effect of USRP cycles on the fatigue performance of titanium alloys. The number of USRP cycles directly affected the surface integrity of TC4 alloy; the surface was smoothest after 1 cycle of USRP, while titanium alloys treated with 12 cycles exhibited the highest surface roughness due to folding defects. All USRP-treated specimens showed higher hardness than the substrate; 1 cycle of USRP induced slight grain refinement, whereas 12 cycles refined grains to nanocrystals with random orientations. illustrates that the fatigue life of USRP-treated titanium alloys was significantly higher than that of the substrate but gradually decreased with increasing USRP cycles. This indicates that USRP treatment shifts the crack initiation site from the material surface to the subsurface, and as the number of cycles increases, the crack initiation site gradually approaches the surface.

The fundamental mechanism underlying this phenomenon resides in the dynamic trade-off between material strengthening and damage induced by Ultrasonic Surface Rolling Processing (USRP), a relationship that evolves with the number of treatment cycles. A single USRP pass produces a relatively smooth surface while introducing substantial compressive residual stresses at the surface. Although the subsurface stress magnitude remains lower than that induced by 12 USRP passes, the absence of stress concentration sources—such as surface wrinkling or microcracks—effectively inhibits the initiation of surface cracks. As a result, the fatigue life of the titanium alloy is significantly enhanced.

### 3.3. Other Surface Deformation Strengthening Methods

Beyond the several commonly employed surface deformation strengthening techniques discussed above, scholars worldwide continue to explore innovative approaches. In the domain of enhancing the fatigue life of titanium alloys via surface deformation strengthening, additional methods include supersonic particle bombardment (SPB) and water jet peening (WJP).

Zhang, Wu et al. [80,93] investigated the effect of supersonic particle bombardment on the fatigue life of titanium alloys. By bombarding the titanium alloy surface with supersonic particles, nanocrystalline structures and compressive residual stresses were introduced, which shifted crack initiation from the surface to the subsurface, thereby enhancing the fatigue life.

Chi, Yao et al. [48,94] examined the influence of water jet peening on the fatigue life of titanium alloys. Water jet peening treatment increased the surface hardness of TA19 relative to the substrate, induced the formation of nanocrystals and high-density dislocations in the surface layer, and transferred the crack initiation site from the material surface to the subsurface—ultimately improving the fatigue life of the material.

The second section of this article presents a comprehensive review of the effects of surface strengthening on the fatigue properties of titanium alloys. It provides an in-depth account of how surface strengthening techniques, including conventional shot peening (CSP), laser shock peening, and ultrasonic surface rolling processing (USRP), can enhance the fatigue performance of titanium alloys. Specifically, shot peening achieves fatigue strength and lifespan improvement of titanium alloys by introducing residual compressive stresses, refining grain structures, and mitigating stress concentrations. Nevertheless, the surface roughness of the treated material is substantially higher than that of the substrate, which may compromise the fatigue performance of titanium alloys. Thus, the enhancement of the fatigue properties of titanium alloys via surface strengthening techniques is essentially attributed to the fact that the positive effects induced by these techniques outweigh their adverse impacts.

## 4. Coating Preparation and Processing Technologies

The surface roughness of titanium alloy is changed by coating, but the fatigue performance of titanium alloy is affected by the preparation process of coating and the properties of the coating itself. The measurement values of fatigue properties of titanium alloy are fatigue life (cycle number) and fatigue strength. Table 3 summarizes detailed information regarding the effects of coating preparation and processing technologies on the fatigue life (cycle number) of titanium alloys under the same fatigue strength.

Table 4 summarizes detailed information regarding the effects of coating preparation and processing technologies on the fatigue strength of titanium alloys under the same cycle number.

### 4.1. Physical Vapor Deposition (PVD) Technology

Physical Vapor Deposition (PVD) is a material surface modification technique that converts solid feedstocks into gaseous particles via physical processes under vacuum conditions, followed by the deposition of these particles onto substrate surfaces to form thin films or coatings [60,112,113]. Key variants of this technology include magnetron sputtering, arc ion plating, and cathodic arc deposition. Magnetron sputtering involves bombarding target materials with high-energy ions to sputter atoms, which then deposit onto the substrate surface. This method exhibits prominent advantages such as high deposition rates and precise compositional control [98,99], and is widely utilized in fields including high-temperature superconducting films and solar cells. Arc ion plating generates high-energy plasma through arc discharge for film deposition, featuring rapid deposition rates and strong interfacial adhesion. It is particularly suited for fabricating coatings with high hardness and excellent wear resistance [26,115], and finds extensive applications in wear-resistant treatment of automotive components and thermal barrier coatings for aero-engines. Cathodic arc deposition produces high-energy ions via arc discharge to ionize metallic materials, which are subsequently deposited onto substrate surfaces. This technique is primarily applied in the manufacturing of dies, molds, and critical wear-resistant components [113,119].

#### 4.1.1. Magnetron Sputtering Technology

Martin et al. [98,99] deposited Cr/CrN coatings on the surface of TC4 alloy via magnetron sputtering. Fractographic analysis of TC4 material reveals that the crack initiation site originates from the coating surface. Fatigue cracks initially form within the brittle CrN layer and subsequently propagate toward the substrate. Although no pores are observed on the coating surface, the cracking within the brittle CrN layer is identified as the primary factor contributing to the degradation of the material’s fatigue performance. Compared with low-stress conditions, the brittle CrN layer under high-stress loading exhibits a significantly shortened crack nucleation period.

Marzich et al. [60] investigated the effect of single plasma electrolytic oxidation (MAO) coatings and MAO/PVD multi-layer coatings on the fatigue performance of TC4 alloy. TC4 with a single MAO coating exhibits the lowest fatigue life, whereas the PVD coating enhances fatigue performance. This improvement is attributed to two key mechanisms: the PVD coating reduces surface roughness by filling the micro-pores inherent in the MAO layer, and the multi-layer structure effectively inhibits fatigue crack propagation. Although the fatigue life of the coated specimens remains lower than that of the uncoated substrate, the fatigue performance is significantly improved compared to specimens with only MAO treatment.

#### 4.1.2. Arc Ion Plating (AIP)

Daiskue et al. [115] investigated the effect of the number of Cr/CrN coating layers (deposited via arc ion plating) on the fatigue performance of TC4 alloy. 3–5 coating layers yield the most significant improvement in the fatigue performance of TC4 alloy, while the performance enhancement of 2-layer coatings is comparable to that of single-layer coatings.

NithyaGnana’s research group [26] investigated the effects of varying bias voltages on coating characteristics and the fatigue performance of titanium alloys. Coatings deposited under low bias voltages exhibited insufficient compressive stress, leading to fatigue crack initiation at the coating surface and thus a lower fatigue life of the alloy compared to the uncoated substrate. In contrast, coatings prepared under high bias voltages possessed high compressive stress, which inhibited surface crack initiation; fatigue cracks instead originated from the subsurface of the substrate, resulting in a significant enhancement of the alloy’s fatigue life.

#### 4.1.3. Cathodic Arc Deposition (CAD)

**Figure 6:**
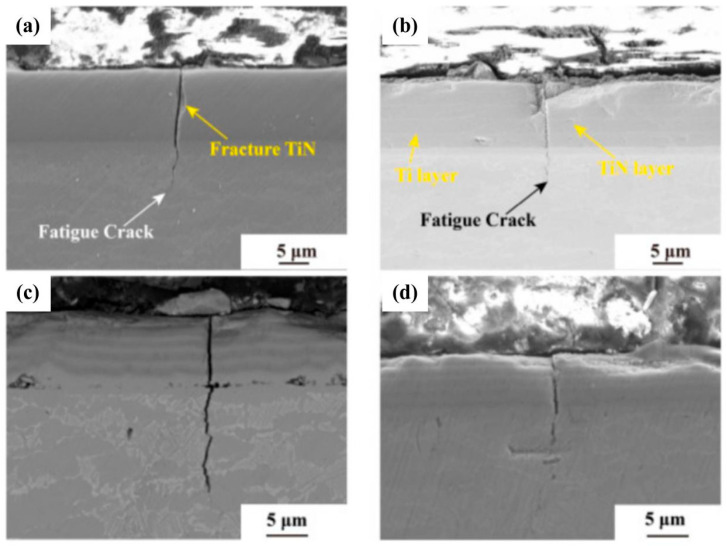
Crack initiation morphology of the single-layer TiN coating (**a**) [119], crack initiation morphology of the 1:1 Ti/TiN coating (**b**) [119], crack initiation morphology of the 3:1 Ti/TiN coating (**c**) [119], crack initiation morphology of the 6:1 Ti/TiN coating (**d**) [119].

Zhang et al. [119] employed TC11 titanium alloy as the substrate, and deposited a single-layer TiN coating as well as three Ti/TiN multilayer coatings with varying thicknesses via cathodic arc deposition (CAD). They investigated the effect of these coatings on the fatigue performance of the titanium alloy. Figure 6 illustrates that surface defects of the coatings induced the early initiation of fatigue cracks, and the rapid penetration of cracks through the coatings accelerated the failure of the substrate. Panel (a) of Figure 6 demonstrates that all coatings significantly degraded the fatigue performance of the titanium alloy, with all fatigue limits being lower than the substrate’s fatigue limit of 855 MPa.

**Figure 7:**
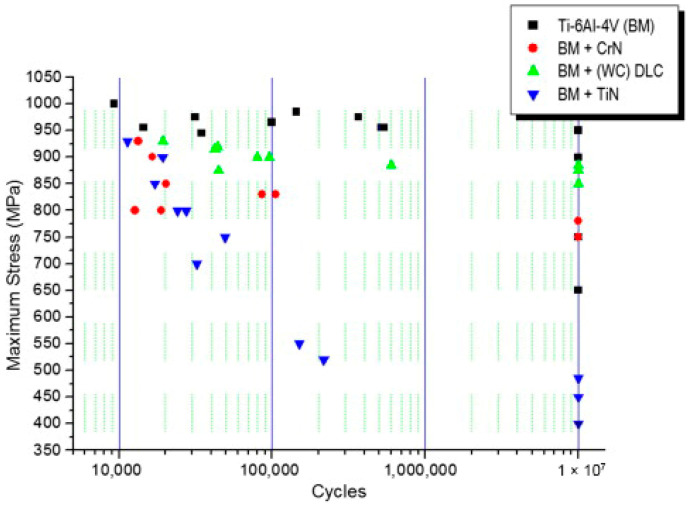
Fatigue life curve of Ti/TiN coatings [113].

Costa’s research group [109,113] investigated the effects of TiN and CrN coatings deposited via cathodic arc deposition (CAD) and coatings prepared by magnetron sputtering on the fatigue performance of TC4 alloy. Panel of Figure 7 demonstrates that all coatings reduced the fatigue strength of the substrate, yet the degree of reduction varied significantly. Coatings fabricated by magnetron sputtering exhibited the optimal performance, with only a 5.5% decrease relative to the substrate—this was attributed to the low defect density inherent to the magnetron sputtering process and the intermediate layer that impeded crack propagation. The fatigue limit of the CrN coating was 750 MPa, where cracks initiated from surface defects of the coating. In contrast, the TiN coating had the lowest fatigue limit of 450 MPa, as pores generated during cathodic arc deposition resulted in the most severe fatigue failure.

### 4.2. Micro-Arc Oxidation

Micro-arc oxidation (MAO) is a surface modification technique for metallic materials. By precisely regulating electrolyte composition and electrical parameters, a ceramic coating with high wear resistance, high strength, and strong adhesion can be formed on the metal surface [54,55,103]. However, MAO coatings typically exhibit a loose and porous surface structure—this inherent feature may induce fatigue cracks to initiate from the coating and propagate to the substrate, thereby degrading the material’s performance. Thus, investigating strategies to mitigate the detrimental effect of MAO coatings on the fatigue performance of titanium alloys is of particular importance [56,104,128].

Shi’s research team [122] investigated the hot salt corrosion fatigue behavior of TC11 titanium alloy, focusing on its performance under ultrasonic surface rolling treatment (USRT), MAO, and their combined treatment. the fatigue limit of the USRT-treated TC11 alloy increased by 10.26%. For the MAO-treated alloy, the formation of a dense barrier layer on its surface contributed to a 47.44% enhancement in fatigue limit. Notably, the combined treatment of USRT and MAO resulted in the greatest improvement of 64.1% in fatigue limit—this synergistic effect of the combined treatment exceeded that of the individual treatments, achieving a significant enhancement in the fatigue performance of the TC11 titanium alloy.

The effect of MAO coating thickness on the fatigue properties of two titanium alloys was investigated by the Apachitei research group [120]. TC4 and Ti6AlNb. The MAO coatings induced a reduction in the high-cycle fatigue strength of both titanium alloys. Notably, the Ti6AlNb alloy with MAO coating exhibited significantly superior fatigue performance compared to the MAO-coated TC4 alloy, and thin MAO coatings exerted a less detrimental effect on fatigue performance than thick ones. The overall degradation of fatigue performance was primarily attributed to the inherent brittleness of the ceramic coating and the porous nature of the coating surface.

Leonardo’s group [123] investigated the effect of MAO treatment on the fatigue performance of Ti6Al7Nb alloy and commercially pure titanium (CP-Ti). Axial fatigue tests revealed no significant changes in the fatigue performance of the two MAO-treated materials: the fatigue limit of Ti6Al7Nb alloy remained at 855 MPa, while that of CP-Ti was maintained at 385 MPa. This negligible change in fatigue performance was primarily attributed to the dense inner layer of the MAO coating, which was free of surface defects.

### 4.3. Additive Manufacturing

Additive Manufacturing (AM), also referred to as 3D printing, is an advanced manufacturing process that constructs three-dimensional components via layer-by-layer material deposition based on 3D model data, leveraging techniques including extrusion, sintering, and melting. This technology offers distinct advantages, such as enabling complex structural design, facilitating rapid prototyping, and allowing precise regulation of geometry and performance [52,53,124]. Among the various AM techniques, laser cladding (LC) stands as a pivotal subfield, defined as a process that deposits alloy or composite layers onto workpiece surfaces through irradiation with high-intensity laser beams. Characterized by rapid cooling rates, strong metallurgical bonding between the cladding layer and substrate, minimal workpiece deformation, and high compatibility with automated systems, LC has been widely adopted in sectors such as aerospace and advanced manufacturing.

Wang et al. [125] employed the laser cladding (LC) process to repair TC17 titanium alloy. During the LC process, residual tensile stresses ranging from 150 to 300 MPa were introduced, and the microhardness of the cladded region was lower than that of the substrate. High-cycle fatigue (HCF) tests revealed that the fatigue limit of the LC-repaired component was 309 MPa, representing a 44.5% reduction compared to the 557 MPa fatigue limit of the as-forged counterpart. Porosity defects were identified as the primary initiation sites for fatigue cracks, while the distinct pit features in the final fracture zone clearly indicated a decrease in the plasticity of the repaired alloy.

Ge et al. [106] employed a hybrid process integrating laser cladding (LC) and laser shock peening for anti-fatigue remanufacturing, aiming to repair surface defects. The surface of the LC zone was free of pores or cracks, with a Widmanstätten structure in its interior, while LSP treatment induced grain refinement. The synergistic combination of these two processes significantly reduced the fatigue crack growth rate (FCGR) and enhanced the fatigue performance of the material.

### 4.4. Other Approaches

Beyond the aforementioned coating preparation and treatment techniques for enhancing the fatigue performance of titanium alloys, scholars worldwide continue to explore innovative solutions, among which the high-velocity oxygen fuel (HVOF) spraying method has been proposed.

Si et al. [107] fabricated a nanostructured coating on the surface of TC6 titanium alloy via the HVOF spraying process. This coating exhibited low porosity and no evident microcracks; its microhardness was approximately 3.2 times that of the substrate. Notably, the coating exerted a negligible impact on the fatigue strength of the substrate, thereby improving the material’s wear resistance while preserving its inherent fatigue performance.

Ma et al. [108] prepared two distinct coatings using HVOF spraying and plasma spraying, respectively, to investigate their effects on fretting fatigue performance. The HVOF-sprayed coating, due to its low toughness, was prone to cracking, which led to a reduction in the material’s fretting fatigue life. In contrast, the plasma-sprayed coating, with its layered microstructure, effectively hindered crack propagation, and its high toughness contributed to an enhancement in the material’s fretting fatigue life.

The third section of the article provides a comprehensive summary of the influence of coating treatments on the fatigue performance of titanium alloys. It elaborates on how surface coating techniques—including physical vapor deposition, micro-arc oxidation, and additive manufacturing—can enhance the fatigue properties of titanium alloys. The impact of coating treatments on fatigue behavior is primarily determined by the type, microstructure, and processing technology of the coatings applied. For example, thin-film metallic glass/ titanium (TFMG/Ti) coatings fabricated via physical vapor deposition effectively inhibit crack initiation, thereby extending the fatigue life of titanium alloys. In contrast, coatings produced by micro-arc oxidation tend to significantly degrade fatigue performance, as these coatings are prone to cracking prematurely relative to the substrate, leading to a reduction in fatigue life.

## 5. Conclusions

Through the research and combing of the literature in the early stage, the following conclusions are obtained on the basis of summarizing and generalizing.

(1) Surface strengthening technology effectively enhances the fatigue strength and prolongs the service life of titanium alloys by introducing residual compressive stress, optimizing grain structure, and alleviating stress concentration. The core principle lies in the fact that the positive effects of surface strengthening technology fully offset the potential negative impacts. The fatigue performance of titanium alloy strengthened by shot peening treatment is improved by 18–30% compared with that of untreated titanium alloy.
(2) The effect of coating treatment on fatigue properties of Ti alloy mainly depends on the type of coating, microstructure and processing technology. Cracks originate from the coating first, and the fatigue properties of Ti alloy are determined by the coating properties. The fatigue performance of titanium alloys is reduced by 26% after applying brittle coatings, while the fatigue life of titanium alloys is increased by 5–26% with coatings prepared by physical vapor deposition (PVD) technology.
(3) Surface strengthening and coating treatment are combined to improve the fatigue performance of the titanium alloy. The coating treatment can optimize the surface roughness increase caused by surface strengthening, and the surface strengthening can reduce the residual stress and make the grain size finer, thus improving the fatigue performance of the titanium alloy. The fatigue strength of the titanium alloy after the joint treatment is 18% higher than that of the untreated substrate.

## 6. Future Perspectives and Outlook

Surface strengthening technology has been widely applied across diverse industrial sectors owing to its capability to enhance the fatigue performance of materials. Current research primarily focuses on surface deformation strengthening and coating preparation treatments, with the aim of improving other properties of materials while retaining or enhancing their fatigue performance—enabling better adaptation to a wide range of service conditions. However, theoretical research on the improvement of titanium alloy fatigue performance via surface strengthening treatments remains insufficient and thus demands in-depth exploration. Future related studies should focus on the following key directions:

1. Surface deformation strengthening treatment exhibits inherent limitations in controlling material surface roughness and plastic deformation extent, which render it challenging to meet the stringent service requirements of the aerospace sector. Thus, the development of strategies to reduce surface roughness and regulate the plastic deformation degree of materials will emerge as a key focus of future research.
2. Coating preparation techniques often introduce pores and cracks on the material surface, which exert detrimental effects on material fatigue performance. Therefore, comprehensive investigations should be conducted on coating selection, preparation methodologies, thickness optimization, and the synergistic effects of multi-coating systems. Such studies will establish a more robust theoretical foundation and practical support for the application of coatings in the field of material fatigue performance.
3. The integration of surface deformation strengthening treatment and coating preparation technology demonstrates significant potential in modulating material fatigue performance. This hybrid approach not only enhances fatigue performance but also improves other properties such as wear resistance, high-temperature resistance, and corrosion resistance. Hence, it merits in-depth and sustained exploration by the academic community.

## Figures and Tables

**Table 1:** Detailed information on the effects of surface strengthening techniques on the fatigue life of titanium alloys.

Surface Treatment	Matrix	Modified Performance Metrics				N_f_				Ref.
		σ_crs_ (MPa)		Ra (µm)		Matrix	After Treatment			
SP	TC4	−660		0.9		<8 × 10^7^	8 × 10^7^			[30]
		−600		3.204		189,574	379,646			[63]
		−450		—		4.7 × 10^6^	4.7 × 10^6^			[64]
		−350		1.29		8 × 10^4^	8 × 10^4^			[65]
	TC11	−782.52		0.8		1.14 × 10^6^	3.47 × 10^6^			[66]
	γ-TiAl	−800		—		4.34 × 10^5^	7.89 × 10^5^			[67]
LSP	TC4	−684		0.883		3.48 × 104	1.81 × 105			[68]
		LSP × 1	−465	—		51,437	LSP × 1		76,126	[69]
		LSP × 2	−646				LSP × 2		110,565	
		LSP-3.6J × 1	−650	—		5.07 × 10^6^	LSP-3.6J × 1		5.99 × 10^6^	[70]
		LSP-3.6J × 3	−720				LSP-3.6J × 3		9.37 × 10^6^	
	Ti17	−700		—		1.05 × 10^5^	2.66 × 10^5^			[71]
		LSP-20J × 3	−712	—		1.68 × 10^5^	LSP-20J × 3		4.05 × 10^5^	[72]
		LSP-30J × 3	−475				LSP-30J × 3		2.90 × 10^5^	
	TC21	LSP × 1	−568	LSP × 1	61.4	14,513	LSP × 1		42,274	[73]
		LSP × 3	−568	LSP × 3	54.8					
		LSP × 5	−790	LSP × 5	74.3					
USP	TC4	USP-2.5 min	−450	USP-2.5 min	1.069	<10,000	USP-5.0min		10,000	[74]
		USP-5.0 min	−550	USP-5.0 min	1.105					
		USP-7.5 min	−620	USP-7.5 min	1.210					
	TA15	−615		0.436		—	6.63 × 10^5^			[75]
USRP	TC4	−1099		0.358		24,700	153,500			[76]
	TC11	−732		0.65		19,314	73,545			[77]
	Ti17	−904.6		0.105		<369,000	369,000			[78]
OTHERS	TA23	—		—		30,370	37,587			[79]
	TA15	SFPB-30 s	−300	SFPB-30 s	5.624	2.67 × 10^4^	SFPB-60 s	1.28 × 10^6^		[80]
		SFPB-60 s	−350	SFPB-60 s	2.011					
		SFPB-90 s	−280	SFPB-90 s	3.646					

Note: SP—conventional shot peening; LSP—laser shock peening; USP—ultrasonic shock peening; SFPB—supersonic fine particle bombardment; USRP—ultrasonic surface rolling processing; σ_crs_—surface residual stress; Ra—surface roughness; N_f_—fatigue cycle count.

**Table 2:** Detailed information on the effects of surface deformation strengthening techniques on the fatigue strength of titanium alloys.

Surface Treatment	Matrix	Modified Performance Metrics				σ_a_ (MPa)					Ref.
		σ_crs_ (MPa)		Ra (µm)		Matrix		After Treatment			
SP	TC4	−660		0.9		542.6		641.1			[31]
		−585		1.286		520		610			[81]
	TI2AlNb	−250		—		170		370			[82]
LSP	TC4	−600		15.0		380		480			[83]
		LSP × 1	−550	LSP × 1	2.2	213		LSP × 1		363	[84]
		LSP × 2	−350								
		LSP × 1	−340	—		216		LSP × 1		264	[85]
		LSP × 2	−420					LSP × 2		306	
		RT-LSP	−575	RT-LSP	1.01	399		RT-LSP		540	[86]
		WLSP	−500	WLSP	1.00			WLSP		568	
	TC11	−760		0.62		438		544			[87]
		LSP × 3	−589.2	LSP × 3	0.68	483.2		LSP × 3		593.6	[88]
		LSP × 5	−610.3	LSP × 5	>0.8						
		LSP × 10	−632.5	LSP × 10	>0.8						
	Ti–2.5Cu	Ti–2.5Cu	−390	Ti–2.5Cu	15	Ti–2.5Cu	425	Ti–2.5Cu		575	[89]
	Ti-54M	Ti-54M	−650	Ti-54M	14	Ti-54M	650	Ti-54M		550	
	LCB	LCB	−450	LCB	11	LCB	650	LCB		800	
USRP	TC4	−1118		0.5		500		600			[90]
		−864.7		0.718		500		610			[91]
		USRP × 1	−963	USRP × 1	0.102	500		USRP × 1	695		[92]
		USRP × 12	−1115	USRP × 12	0.163			USRP × 12	640		
	TC11	SFPB	−196	SFPB	5.531	<500		500			[93]
	TA19	WJP1	−614	WJP1	0.366	393.33		WJP1	446.67		[94]
		WJP2	−647	WJP2	0.360						

Note: SP—conventional shot peening; LSP—laser shock peening; USP—ultrasonic shock peening; SFPB—supersonic fine particle bombardment; USRP—ultrasonic surface rolling processing; σ_crs_—surface residual stress; Ra—surface roughness; N_f_—fatigue cycle count.

**Table 3:** Detailed Information on the effects of coating preparation and processing technologies on the fatigue life of titanium alloys.

Coating Technology	Matrix	Coating	Coating Thickness (µm)					N_f_			Ref
					Ra (µm)			Matrix	After Treatment		
PVD	TC4	Cr/CrN	—		—			848,334	23,042		[98]
			—		—			8.8 × 10^5^	2.3 × 10^4^		[99]
		TiN	TiN	0.2	TiN		0.035	2.2 × 10^5^	TiN	1.0 × 10^6^	[100]
		TFMG/Ti	TFMG/Ti	0.21	TFMG/Ti		0.029		TFMG/Ti	3.7 × 10^6^	
	TC17	TiN	TiN	3	—			5.22 × 10^5^	TiN	6.56 × 10^5^	[101]
		TiN/Ti	TiN/Ti	6					TiN/Ti	1.986 × 10^6^	
	Ti46Al8Nb	TiN/Ti	—		—			—	TiN/Ti × 1	280	[102]
									TiN/Ti × 10	300	
MAO	TC4	TiO_2_	TiO_2_	10.9	TiO_2_		1.7	4 × 10^5^	TiO_2_	1 × 10^5^	[40]
			TiO_2_ + USRP × 1	11	TiO_2_ + USRP × 1		1.5		TiO_2_ + USRP × 1	3 × 10^5^	
			TiO_2_ + USRP × 12	11.9	TiO_2_ + USRP × 12		1.6		TiO_2_ + USRP × 12	8 × 10^5^	
			TiO_2_	20	TiO_2_		3.098	—	TiO_2_	2.66 × 10^4^	[103]
			TiO_2_ + USRP	20	TiO_2_ + USRP		2.553		TiO_2_ + USRP	3.72 × 10^5^	
			TiO_2_	50	TiO_2_		5.503		TiO_2_	1.55 × 10^4^	
			TiO_2_ + USRP	50	TiO_2_ + USRP		4.682		TiO_2_ + USRP	4.24 × 10^4^	
	TA15		TiO_2_-10 min	13	—			2.08 × 10^6^	TiO_2_-10 min	3.52 × 10^4^	[104]
			TiO_2_-30 min	25					TiO_2_-30 min	3 × 10^4^	
AM	TC4	—	—		—			4.6 × 10^4^	5.5 × 10^6^		[105]
		LC	—		—			22,283	58,835		[106]
OTHERS	TC6	HVOF	—		—			1 × 10^7^	<1 × 10^7^		[107]
	TC4	HVOF	—		HVOF	1.0		5 × 10^4^	HVOF	2.5 × 10^4^	[108]
		PS			PS	>1.0			PS	6 × 10^4^	

Note: physical vapor deposition (PVD); magnetron sputtering (MS); arc ion plating (AIP); cathodic arc deposition (CAD); micro-arc oxidation (MAO); additive manufacturing (AM); high-velocity oxygen fuel spraying (HVOF); atmospheric plasma spraying (APS); plasma spraying (PS); laser powder bed fusion (LPBF); laser cladding coating (LC).

**Table 4:** Detailed Information on the effects of coating preparation and processing technologies on the fatigue life of titanium alloys.

Coating Preparation and Processing Techniques	Matrix	Coating	Coating Thickness (µm)		Modified Performance Metrics		N_f_			Ref.
					Ra (µm)		Matrix	After Treatment		
PVD	TC4	TiN	4		1.06		900	450		[109]
		TiZrN/TiZr	20		0.44		512.5	487.5		[110]
		TiN/Ti	11.5		2.13		251	396.8		[111]
		CrAlN	CrAlN × 1	4.5	—		510	CrAlN × 1	315	[112]
			CrAlN × 2	9				CrAlN × 2	<315	
		TiN	TiN	10				TiN	<315	
		TiN	TiN	4	TiN	1.06	900	TiN	450	[113]
		CrN	CrN	4	CrN	1.08		CrN	750	
		DLC	DLC	2.4	DLC	0.57		DLC	850	
		CrN	CrN	3.02	—		700	CrN	500	[114]
		TiN	TiN	3.03				TiN	550	
		Cr/CrN	Cr/CrN × 1	2	Cr/CrN × 1	0.195	650	Cr/CrN × 1	650	[115]
			Cr/CrN × 2	2	Cr/CrN × 2	0.176		Cr/CrN × 2	650	
			Cr/CrN × 3	2	Cr/CrN × 3	0.177		Cr/CrN × 3	800	
			Cr/CrN × 4	2	Cr/CrN × 4	0.136		Cr/CrN × 4	800	
			Cr/CrN × 5	2	Cr/CrN × 5	0.164		Cr/CrN × 5	800	
		TiN	TiN	1	TiN	0.3	475	TiN	500	[116]
		TiN/AlN	TiN/AlN	1	TiN/AlN	0.3		TiN/AlN	587.5	
		TiN	TiN	5	—		510	TiN	290	[117]
		TiN-Cr	TiN-Cr	5.07				TiN-Cr	400	
	TC11	TiN	10		—		582.5	547.5		[118]
	TC11	TiN/Ti	TiN	10–12	TiN	4–8	855	TiN	550	[119]
			TiN/Ti × 6	10–12	TiN/Ti × 6	4–8		TiN/Ti × 6	525	
			TiN/Ti × 3	10–12	TiN/Ti × 3	4–8		TiN/Ti × 3	500	
			TiN/Ti × 1	10–12	TiN/Ti × 1	4–8		TiN/Ti × 1	400	
MAO	TC4	TiO_2_	—		—		580	278.4		[120]
			—		—		845	845		[121]
	TC11		—		—		390	575		[122]
	Ti6Al7Nb		—		0.13		855	855		[123]
AM	TC4	LDED	—		0.4		404.3	452.6		[124]
	TC17	LC	—		0.4		557	309		[125]
OTHERS	TC4	HVOF	150		2.77		900	400		[126]
		APS	—		—		615	620		[127]

Note: physical vapor deposition (PVD); magnetron sputtering (MS); arc ion plating (AIP); cathodic arc deposition (CAD); micro-arc oxidation (MAO); additive manufacturing (AM); high-velocity oxygen fuel spraying (HVOF); atmospheric plasma spraying (APS); plasma spraying (PS); laser powder bed fusion (LPBF); laser cladding coating (LC).

## Data Availability

No new data were created or analyzed in this study. Data sharing is not applicable to this article.

## Supplementary Materials

The following supporting information can be downloaded at: https://www.mdpi.com/article/10.3390/ma19081511/s1, PRISMA checklist.

## References

[1] Li B., Xia Y., Li W., Jiang C., Chen H. (2026). The critical assessment for the fatigue life of additive manufacturing TC4 and Inconel 718 notched component. Theor. Appl. Fract. Mech..

[2] Li Z., Shen X., Hu H., Mei C., Kui Z., He Z., Li Z., Jiang Y. (2025). Ultralight biomedical TC4-Cu alloy achieveing superior mechanical properties and osseointegration. Mater. Des..

[3] Lou K., Zhu M., Yuan Y., Guo S. (2025). A research on alternating current corrosion of TC4 alloy with different microstructures in ocean environment. Surf. Interfaces.

[4] Yu W., Yin Y., Zhou J., Li W., Zuo J., Lin J., Feng X. (2022). High cycle fatigue behaviors and deformation mechanisms in Ti47Al2Cr2Nb alloy at room temperature and 700 °C. J. Mater. Res. Technol..

[5] Sun C., Li Y., Huang R., Wang L., Liu J., Zhou L., Duan G. (2020). Crack initiation mechanism and fatigue life of titanium alloy Ti–6Al–2Sn–2Zr–3Mo-X: Effects of stress ratio and loading frequency. Mater. Sci. Eng. A.

[6] Rui S.-S., Liu J., Zhang Y., Chen P., Sun C. (2025). Effects of stress state on fatigue crack initiation and fatigue performance of Ti-6Al-3Nb-2Zr-1Mo titanium alloy used in deep-sea hull structure. Eng. Fract. Mech..

[7] Guo Y., Dong R., Li C., Yang L., Hou H., Zhao Y. (2025). Microstructural evolution and mechanical properties of an (α + β) titanium alloy subjected to cyclic multi-directional forging. Mater. Charact..

[8] Heinz S., Eifler D. (2016). Crack initiation mechanisms of Ti6Al4V in the very high cycle fatigue regime. Int. J. Fatigue.

[9] Lu Y., Lu Y., Dang H., Li X., Zhang S., He M., Ran G., Yi X., Hu R., Wang H. (2025). Damage mechanism study of TC17 titanium alloy during high-cycle fatigue crack initiation and propagation. J. Alloys Compd..

[10] Yu Y., Gong J., Fang X., Zhou L., He W., Zhou L., Cai Z. (2023). Comparison of surface integrity of GH4169 superalloy after high-energy, low-energy, and femtosecond laser shock peening. Vacuum.

[11] Ren J.Q., Li L., Wang Q., Xin C., Gao Q., Li J.C., Xue H.T., Lu X.F., Tang F.L. (2024). Effect of environmental media on the growth rate of fatigue crack in TC4 titanium alloy: Seawater and air. Corros. Sci..

[12] Zhang C., Luo X., Han S., Wu Z., Zou H., Hu R., Luo W. (2025). Effect of high-temperature long-term aging/annealing treatment on the microstructure and mechanical properties of bimodal heterogeneous TiBw/TC4 composites. Mater. Charact..

[13] Zhang Y., Zha X., Feng K., Li Y., Li J., Hu J., Zu Z., Zhang C., Wang C., Dou C. (2026). Oxidation and crack propagation in the TC4 titanium alloy under high-speed transient impact-rubbing leading to Pre-Ignition. Eng. Fail. Anal..

[14] Song B., Wang X., Xie L., Xiang J., Umer U., Abidi M.H., Almutairi Z. (2024). The effect of surface roughness and microstructure on fretting fatigue properties of TC21 based on hierarchical multiscale modeling. J. Mater. Res. Technol..

[15] Wu G.Q., Li Z., Sha W., Li H.H., Huang L.J. (2014). Effect of fretting on fatigue performance of Ti-1023 titanium alloy. Wear.

[16] Lei Y.-w., Ma G.-l., Liu Y., Zhao W., Wu H.-p., Li X.-f. (2024). Vibration fatigue properties and deterioration mechanism of diffusion bonded TC4 titanium alloy. Trans. Nonferrous Met. Soc. China.

[17] Chi W., Wang W., Xu W., Li G., Chen X., Sun C. (2022). Effects of defects on fatigue behavior of TC17 titanium alloy for compressor blades: Crack initiation and modeling of fatigue strength. Eng. Fract. Mech..

[18] Luo S., Nie X., Zhou L., Li Y., He W. (2018). High Cycle Fatigue Performance in Laser Shock Peened TC4 Titanium Alloys Subjected to Foreign Object Damage. J. Mater. Eng. Perform..

[19] Kahlin M., Ansell H., Basu D., Kerwin A., Newton L., Smith B., Moverare J.J. (2020). Improved fatigue strength of additively manufactured Ti6Al4V by surface post processing. Int. J. Fatigue.

[20] Lainé S.J., Knowles K.M., Doorbar P.J., Cutts R.D., Rugg D. (2017). Microstructural characterisation of metallic shot peened and laser shock peened Ti–6Al–4V. Acta Mater..

[21] Zhang Z., Yang Z., He G. (2023). Alleviating the adverse influence of nitride coating on the fatigue performance of Ti6Al4V by Ni alloying. J. Mater. Res. Technol..

[22] Meng X.-k., Zhou J.-z., Su C., Huang S., Luo K.-y., Sheng J., Tan W. (2017). Residual stress relaxation and its effects on the fatigue properties of Ti6Al4V alloy strengthened by warm laser peening. Mater. Sci. Eng. A.

[23] Li G., Chi W., Wang W., Liu X., Tu H., Long X. (2024). High cycle fatigue behavior of additively manufactured Ti-6Al-4V alloy with HIP treatment at elevated temperatures. Int. J. Fatigue.

[24] Yoder G., Cooley L., Crooker T. (1979). Quantitative analysis of microstructural effects on fatigue crack growth in widmanstätten Ti-6A1-4V and Ti-8Al-1Mo-1V. Eng. Fract. Mech..

[25] Srinivasan S., Garcia D.B., Gean M.C., Murthy H., Farris T.N. (2009). Fretting fatigue of laser shock peened Ti–6Al–4V. Tribol. Int..

[26] Sivagnanam Chandra N.P., Otsuka Y., Mutoh Y., Yamamoto K. (2020). Fatigue strength and mechanism of Ti6242S titanium alloy with TiAlN coating deposited under various bias voltages. Int. J. Fatigue.

[27] Caron I., De Monicault J., Gras R. (2001). Influence of surface-coatings on titanium-alloy resistance to fretting fatigue in cryogenic environment. Tribol. Int..

[28] Cassar G., Avelar-Batista Wilson J.C., Banfield S., Housden J., Fenech M., Matthews A., Leyland A. (2011). Evaluating the effects of plasma diffusion processing and duplex diffusion/PVD-coating on the fatigue performance of Ti–6Al–4V alloy. Int. J. Fatigue.

[29] Peters J., Ritchie R. (2001). Foreign-object damage and high-cycle fatigue of Ti–6Al–4V. Mater. Sci. Eng. A.

[30] Mower T.M. (2014). Degradation of titanium 6Al–4V fatigue strength due to electrical discharge machining. Int. J. Fatigue.

[31] Shi H., Liu D., Pan Y., Zhao W., Zhang X., Ma A., Liu B., Hu Y., Wang W. (2021). Effect of shot peening and vibration finishing on the fatigue behavior of TC17 titanium alloy at room and high temperature. Int. J. Fatigue.

[32] Zhu W.-g., Ma C., Zhang C.-h., Hu K., Zeng X.-k. (2023). Fatigue crack propagation behavior in Ti−6Al−4V alloy with surface gradient structure fabricated by high-energy shot peening. Trans. Nonferrous Met. Soc. China.

[33] Xie L., Wen Y., Zhan K., Wang L., Jiang C., Ji V. (2016). Characterization on surface mechanical properties of Ti–6Al–4V after shot peening. J. Alloys Compd..

[34] Lan L., Jin X., Gao S., He B., Rong Y. (2020). Microstructural evolution and stress state related to mechanical properties of electron beam melted Ti-6Al-4V alloy modified by laser shock peening. J. Mater. Sci. Technol..

[35] Nalla R.K., Altenberger I., Noster U., Liu G.Y., Scholtes B., Ritchie R.O. (2003). On the influence of mechanical surface treatments—Deep rolling and laser shock peening—On the fatigue behavior of Ti–6Al–4V at ambient and elevated temperatures. Mater. Sci. Eng. A.

[36] Hu W., Yi M. (2024). Predicting tensile behavior and fatigue life of laser shock peened titanium alloy by crystal plasticity model. Int. J. Fatigue.

[37] Tong Z., Ren X., Ren Y., Dai F., Ye Y., Zhou W., Chen L., Ye Z. (2018). Effect of laser shock peening on microstructure and hot corrosion of TC11 alloy. Surf. Coat. Technol..

[38] Luo X., Xu Z., Tian K., Wang Y., Wang X., Wang K., Yu Y., Ye C., Dang N. (2025). Effect of overlap pattern on the residual stress, surface morphology and fatigue properties of Ti-6Al-4V alloy by multiple laser shock peening. Opt. Laser Technol..

[39] Wang H., Song G., Tang G. (2016). Evolution of surface mechanical properties and microstructure of Ti 6Al 4V alloy induced by electropulsing-assisted ultrasonic surface rolling process. J. Alloys Compd..

[40] Ao N., Liu D., Zhang X., Liu C. (2019). Enhanced fatigue performance of modified plasma electrolytic oxidation coated Ti-6Al-4V alloy: Effect of residual stress and gradient nanostructure. Appl. Surf. Sci..

[41] Nie X., He W., Zhou L., Li Q., Wang X. (2014). Experiment investigation of laser shock peening on TC6 titanium alloy to improve high cycle fatigue performance. Mater. Sci. Eng. A.

[42] Nie X., He W., Cao Z., Song J., Li X., Pang Z., Yan X. (2021). Experimental study and fatigue life prediction on high cycle fatigue performance of laser-peened TC4 titanium alloy. Mater. Sci. Eng. A.

[43] Sun R., Che Z., Cao Z., Zou S., Wu J., Guo W., Zhu Y. (2020). Fatigue behavior of Ti-17 titanium alloy subjected to different laser shock peened regions and its microstructural response. Surf. Coat. Technol..

[44] Lin B., Lupton C., Spanrad S., Schofield J., Tong J. (2014). Fatigue crack growth in laser-shock-peened Ti–6Al–4V aerofoil specimens due to foreign object damage. Int. J. Fatigue.

[45] Luo X., Wang Y., Dang N., Tian K., Wang K., Zhao C., Zha X., Wang X., Yu Y. (2022). Gradient microstructure and foreign-object-damaged fatigue properties of Ti6Al4V titanium alloy processed by the laser shock peening and subsequent shot peening. Mater. Sci. Eng. A.

[46] Li J., Zhou J., Liu L., Feng A., Huang S., Meng X. (2021). High-cycle bending fatigue behavior of TC6 titanium alloy subjected to laser shock peening assisted by cryogenic temperature. Surf. Coat. Technol..

[47] Yu Z., Yu K., Pan X., Yu Y., Liang X., Zhou L. (2024). High-precision control of microstructure and mechanical properties of Ti–6Al–4V thin-walled titanium alloy components by laser peening without coating. J. Mater. Res. Technol..

[48] Yao S.-L., Wang G.-Y., Yu H., Wang J., Li K.-S., Liu S., Zhang X.-C., Tu S.-T. (2022). Influence of submerged micro-abrasive waterjet peening on surface integrity and fatigue performance of TA19 titanium alloy. Int. J. Fatigue.

[49] Sivagnanam Chandra N.P., Otsuka Y., Mutoh Y., Yamamoto K. (2020). Effect of coating thickness on fatigue behavior of TiAlN coated Ti-alloys. Int. J. Fatigue.

[50] Zhou K., Liu D., Zhang X., Liu Y., Li M., Wu J., Yang Z. (2025). TiAlTaSiN/TiAlTaSi multilayer coatings for enhancing hot salt corrosion fatigue resistance of TC11 Alloy. Corros. Sci..

[51] Guo N., Yu J., Zhou Q., Wang J., Xiao G., Tang B., Zhang Z. (2025). Enhancing low-cycle fatigue performance of high-efficient laser additively manufactured TC11 titanium alloy: Mechanisms of cyclic softening/hardening and microstructural refinement. J. Mater. Res. Technol..

[52] Ghosh A., Ahmed S., Reddy S.T., Shankar G. (2025). Dwell-fatigue behaviour of additively manufactured Ti6242 alloy via LPBF and HIPPING. Int. J. Fatigue.

[53] Zhang Y., Liu Z., Yang C., Wang X., Sun M., Chen S., Xu G. (2025). Microstructure and wear behavior of laser-cladded WC matrix composite coatings with different TiC/Ni contents on titanium alloys. Ceram. Int..

[54] Li P., Yang Z., He B., Wang N., Chen Y., Zhao Q., Kang Y., Zhang X., Zhao Y. (2023). Effect of local ‘over-growth’ on fracture behaviors of coated titanium fiber fabricated by plasma electrolytic oxidation. Surf. Interfaces.

[55] Chen S., Wu J., Tu J., Wang H., Xiong X., Hu Q., Zou J., Zeng X. (2016). Effect of plasma electrolytic oxidation treatment on the mechanical properties of a Zr–Cu–Ni–Ti–Al bulk metallic glass. Mater. Sci. Eng. A.

[56] Wang X.-m., Zhang F.-q. (2023). Effects of soft sparking on micro/nano structure and bioactive components of microarc oxidation coatings on selective laser melted Ti6Al4V alloy. Surf. Coat. Technol..

[57] Luo J., Tang W., Cui S. (2023). The effect of laser shock peening on the microstructure and wear resistance of micro-arc oxidation coatings on TC4 alloy. J. Mater. Res. Technol..

[58] Ao N., Liu D., Zhang X., Fan K., Shi H., Liu Z., Liu C. (2019). The effect of residual stress and gradient nanostructure on the fretting fatigue behavior of plasma electrolytic oxidation coated Ti–6Al–4V alloy. J. Alloys Compd..

[59] Tong Z.P., Ren X.D., Zhou W.F., Adu-Gyamfi S., Chen L., Ye Y.X., Ren Y.P., Dai F.Z., Yang J.D., Li L. (2019). Effect of laser shock peening on wear behaviors of TC11 alloy at elevated temperature. Opt. Laser Technol..

[60] Ebrahimi M., Kermanpur A., Kharaziha M., Bagherifard S. (2024). Engineering of multilayered coating on additively manufactured Ti-6Al-4V porous implants to promote tribological and fatigue performances. Surf. Coat. Technol..

[61] Wu J., Liu D., Zhang X., Liu Y., Yang Z., Xiang J. (2025). Enhancing high-temperature fatigue resistance of TC11 titanium alloy through combined plasma zirconizing and ultrasonic surface rolling. Int. J. Fatigue.

[62] Gavalec M., Barenyi I., Krbata M., Kohutiar M., Balos S., Pecanac M. (2023). The Effect of Rotary Friction Welding Conditions on the Microstructure and Mechanical Properties of Ti6Al4V Titanium Alloy Welds. Materials.

[63] Yang Q., Zhou W., Gai P., Zhang X., Fu X., Chen G., Li Z. (2017). Investigation on the fretting fatigue behaviors of Ti-6Al-4V dovetail joint specimens treated with shot-peening. Wear.

[64] Pant B.K., Pavan A.H.V., Prakash R.V., Kamaraj M. (2016). Effect of laser peening and shot peening on fatigue striations during FCGR study of Ti6Al4V. Int. J. Fatigue.

[65] Wang Q., Hu T., Ren Y., Xu P., Wu Y., Zhi X., Lin J., Qin G. (2025). Ultrasonic fatigue performance of the shot-peened TC4 titanium Alloy: Improved or deteriorated?. Mater. Sci. Eng. A.

[66] Ren X., Wang Z., An R. (2025). A promising approach to enhance fatigue life of TC11 titanium alloy: Low dislocation density and surface grain refinement induced by electropulsing. J. Mater. Sci. Technol..

[67] Breuner C., Guth S., Gall E., Swadźba R., Gibmeier J., Heilmaier M. (2021). Influence of Shot Peening on the Isothermal Fatigue Behavior of the Gamma Titanium Aluminide Ti-48Al-2Cr-2Nb at 750 °C. Metals.

[68] Luo X., Dang N., Wang X. (2021). The effect of laser shock peening, shot peening and their combination on the microstructure and fatigue properties of Ti-6Al-4V titanium alloy. Int. J. Fatigue.

[69] Wang Z., Zhou W., Luo K., Lu H., Lu J. (2023). Strengthening mechanism in thermomechanical fatigue properties of Ti6Al4V titanium alloy by laser shock peening. Int. J. Fatigue.

[70] Wang B., Cheng L., Li D. (2022). Study on very high cycle fatigue properties of forged TC4 titanium alloy treated by laser shock peening under three-point bending. Int. J. Fatigue.

[71] Sun R., Keller S., Zhu Y., Guo W., Kashaev N., Klusemann B. (2021). Experimental-numerical study of laser-shock-peening-induced retardation of fatigue crack propagation in Ti-17 titanium alloy. Int. J. Fatigue.

[72] Sun R., Li L., Guo W., Peng P., Zhai T., Che Z., Li B., Guo C., Zhu Y. (2018). Laser shock peening induced fatigue crack retardation in Ti-17 titanium alloy. Mater. Sci. Eng. A.

[73] Song B., Wang X., Xie L., Xiang J., Zou X., Zou S. (2024). Effect of laser shock peening on the surface integrity and fretting fatigue properties of high-strength titanium alloy TC21. J. Mater. Res. Technol..

[74] Kumar S., Chattopadhyay K., Singh V. (2020). Optimization of the Duration of Ultrasonic Shot Peening for Enhancement of Fatigue Life of the Alloy Ti-6Al-4V. J. Mater. Eng. Perform..

[75] Wu B., Huang J., Yang G., Ren Y., Zhou S., An D. (2022). Effects of ultrasonic shot peening on fatigue behavior of TA15 titanium alloy fabricated by laser melting deposition. Surf. Coat. Technol..

[76] Fan K., Liu D., Zhang X., Liu D., Zhao W., Yang J., Ma A., Li M., Qi Y., Xiang J. (2022). Effect of residual stress induced by ultrasonic surface rolling on fretting fatigue behaviors of Ti-6Al-4V alloy. Eng. Fract. Mech..

[77] Fan K., Liu D., Zhou K., Liu Y., Zhang H., Zhang X., Li Y., Li M., Li Y., Abdel Wahab M. (2024). Enhanced fretting fatigue strength of TC11 titanium alloy using laser-assisted ultrasonic surface rolling process. Tribol. Int..

[78] Tan L., Tang W., Wang M., Zhang Y., Yao C. (2025). Studies on surface integrity and fatigue performance of Ti-17 alloy induced by ultrasonic surface rolling process. Surf. Coat. Technol..

[79] Chen Z., Peng C., Zuo Y. (2024). Effects of lubrication conditions on cold expansion fatigue strengthening performances of titanium alloy open hole structures. Ocean Eng..

[80] Zhang L., Wang Q., Zhang J., Xiong Y., Yao H. (2025). Effect of gradient nanostructure prepared by supersonic fine particle bombardment on the microstructure and corrosion fatigue property of TA15 titanium alloy. Surf. Coat. Technol..

[81] Ji D., Chen H., Zhang J., Su K., Chen X. (2025). Influence of micro-shot peening and traditional shot peening on fatigue performance and fracture behaviors of Ti-6Al-4V alloy. Int. J. Fatigue.

[82] Chen Y.X., Wang J.C., Gao Y.K., Feng A.H. (2019). Effect of shot peening on fatigue performance of Ti2AlNb intermetallic alloy. Int. J. Fatigue.

[83] Altenberger I., Nalla R.K., Sano Y., Wagner L., Ritchie R.O. (2012). On the effect of deep-rolling and laser-peening on the stress-controlled low- and high-cycle fatigue behavior of Ti–6Al–4V at elevated temperatures up to 550 °C. Int. J. Fatigue.

[84] Gu H., Yan P., Jiao L., Chen S., Song Y., Zou S., Wang X. (2023). Effect of laser shock peening on boring hole surface integrity and conformal contact fretting fatigue life of Ti-6Al-4 V alloy. Int. J. Fatigue.

[85] Zhang X.C., Zhang Y.K., Lu J.Z., Xuan F.Z., Wang Z.D., Tu S.T. (2010). Improvement of fatigue life of Ti–6Al–4V alloy by laser shock peening. Mater. Sci. Eng. A.

[86] Feng X., Pan X., He W., Liu P., An Z., Zhou L. (2021). Improving high cycle fatigue performance of gas tungsten arc welded Ti6Al4V titanium alloy by warm laser shock peening. Int. J. Fatigue.

[87] Luo S., Zhou L., Nie X., Li Y., He W. (2019). The compound process of laser shock peening and vibratory finishing and its effect on fatigue strength of Ti-3.5Mo-6.5Al-1.5Zr-0.25Si titanium alloy. J. Alloys Compd..

[88] Nie X., He W., Zang S., Wang X., Zhao J. (2014). Effect study and application to improve high cycle fatigue resistance of TC11 titanium alloy by laser shock peening with multiple impacts. Surf. Coat. Technol..

[89] Maawad E., Sano Y., Wagner L., Brokmeier H.G., Genzel C. (2012). Investigation of laser shock peening effects on residual stress state and fatigue performance of titanium alloys. Mater. Sci. Eng. A.

[90] Ao N., Liu D., Zhang X., Wu S. (2023). Improved fretting fatigue mechanism of surface-strengthened Ti-6Al-4V alloy induced by ultrasonic surface rolling process. Int. J. Fatigue.

[91] Liu C., Liu D., Zhang X., Liu D., Ma A., Ao N., Xu X. (2019). Improving fatigue performance of Ti-6Al-4V alloy via ultrasonic surface rolling process. J. Mater. Sci. Technol..

[92] Liu C., Liu D., Zhang X., He G., Xu X., Ao N., Ma A., Liu D. (2019). On the influence of ultrasonic surface rolling process on surface integrity and fatigue performance of Ti-6Al-4V alloy. Surf. Coat. Technol..

[93] Wu Y., Xiong Y., Liu W., Chen Z., Zhang X., Wang S., Cao W. (2021). Effect of supersonic fine particle bombardment on microstructure and fatigue properties of Ti-6.5Al-3.5Mo-1.5Zr-0.3Si titanium alloy at different temperatures. Surf. Coat. Technol..

[94] Chi Y.-X., Yao S.-L., Cheng H.-Y., Zhu X.-H., Liu J.-W., Liu C.-L., Wang N., Zhang C.-C., Zhang X.-C. (2025). Bilateral submerged abrasive waterjet peening improved high-temperature fatigue strength of titanium alloy thin-walled simplified blades. J. Manuf. Process..

[95] Kumar S., Pandey V., Chattopadhyay K., Singh V. (2018). Surface Nanocrystallization Induced by Ultrasonic Shot Peening and Its Effect on Corrosion Resistance of Ti–6Al–4V Alloy. Trans. Indian Inst. Met..

[96] Pereira Dos Santos M., Farias A., Bordinassi E.C., Delijaicov S. (2026). Experimental data on the generation of compressive residual stresses via shot peening and subsequent heat treatment in SAE 5160 and DIN 51CrV4 spring steels. Data Brief.

[97] Chen L., Xie J., Li Y., Yu G., Zhang X., Ren X. (2024). Effect of laser/ultrasonic shock peening on the microstructure and mechanical properties of nickel-based superalloys prepared by Powder Bed Fusion Laser Beam (PBF-LB). J. Mater. Res. Technol..

[98] Fernandes M.F., Velloso V.M.d.O., Callisaya E.S., Voorwald H.J.C. (2024). Cr/CrN coating effect on the fatigue crack nucleation period of Ti-6Al-4V alloy. Mater. Today Commun..

[99] Fernandes M.F., de Oliveira Velloso V.M., Voorwald H.J.C. (2022). Cr/CrN multilayer coating effect on the surface integrity of Ti-6Al-4V alloy under fatigue loadings. Procedia CIRP.

[100] Lee C.M., Chu J.P., Chang W.Z., Lee J.W., Jang J.S.C., Liaw P.K. (2014). Fatigue property improvements of Ti–6Al–4V by thin film coatings of metallic glass and TiN: A comparison study. Thin Solid Film..

[101] Zhang X.H., Liu D.X., Tan H.B., Wang X.F. (2009). Effect of TiN/Ti composite coating and shot peening on fretting fatigue behavior of TC17 alloy at 350 °C. Surf. Coat. Technol..

[102] Zhou Y., Rao G.B., Wang J.Q., Zhang B., Yu Z.M., Ke W., Han E.H. (2011). Influence of Ti/TiN bilayered and multilayered films on the axial fatigue performance of Ti46Al8Nb alloy. Thin Solid Film..

[103] Wang S., Yu T., Pang Z., Liu X., Shi C., Du N. (2024). Improving the fatigue resistance of plasma electrolytic oxidation coated titanium alloy by ultrasonic surface rolling pretreatment. Int. J. Fatigue.

[104] Wang Y.M., Zhang P.F., Guo L.X., Ouyang J.H., Zhou Y., Jia D.C. (2009). Effect of microarc oxidation coating on fatigue performance of Ti–Al–Zr alloy. Appl. Surf. Sci..

[105] Yoshinaka F., Nakamura T., Takaku K. (2016). Effects of vacuum environment on small fatigue crack propagation in Ti–6Al–4V. Int. J. Fatigue.

[106] Ge M.Z., Tang Y., Zhang Y.K., Wang Y. (2022). Enhancement in fatigue property of Ti-6Al-4V alloy remanufactured by combined laser cladding and laser shock peening processes. Surf. Coat. Technol..

[107] Si C., Li S., Zhao L., Xu S., Chen S. (2024). Effect of HVOF–sprayed nanostructured WC–10Co–4Cr coating on sliding wear and tensile–tensile fatigue properties of TC6 titanium alloy. Int. J. Refract. Met. Hard Mater..

[108] Ma A., Liu D., Zhang X., He G., Liu D., Liu C., Xu X. (2020). The fretting fatigue performance of Ti–6Al–4V alloy influenced by microstructure of CuNiIn coating prepared via thermal spraying. Tribol. Int..

[109] Costa M.Y.P., Cioffi M.O.H., Venditti M.L.R., Voorwald H.J.C. (2010). Fatigue fracture behavior of Ti-6Al-4V PVD coated. Procedia Eng..

[110] Liu Y., Liu D., Ma A., Yang Z., Wu J., Zhou K., Li M., Zhang X. (2025). Gradient TiZrN coating and deformation-enhanced pretreatment to improve the anti-solid particle erosion and fatigue performance of Ti-6Al-4V alloy. Eng. Fail. Anal..

[111] Cao X., He W., Liao B., He G., Jiao Y., Huang D., Wang S. (2020). Effect of TiN/Ti coating combined with laser shock peening pre-treatment on the fatigue strength of Ti-6Al-4V titanium alloy. Surf. Coat. Technol..

[112] Bai Y., Xi Y., Gao K., Yang H., Pang X., Yang X., Volinsky A.A. (2019). Brittle coating effects on fatigue cracks behavior in Ti alloys. Int. J. Fatigue.

[113] Costa M.Y.P., Venditti M.L.R., Cioffi M.O.H., Voorwald H.J.C., Guimarães V.A., Ruas R. (2011). Fatigue behavior of PVD coated Ti–6Al–4V alloy. Int. J. Fatigue.

[114] Zhang Z., Chen J., He G., Yang G. (2019). Fatigue and Mechanical Behavior of Ti-6Al-4V Alloy with CrN and TiN Coating Deposited by Magnetic Filtered Cathodic Vacuum Arc Process. Coatings.

[115] Yonekura D., Fujita J., Miki K. (2015). Fatigue and wear properties of Ti–6Al–4V alloy with Cr/CrN multilayer coating. Surf. Coat. Technol..

[116] Wang J., Guo T., Chen Y., Wang X., Geng P., Gao K., Pang X. (2023). Significant improvement in fatigue life of titanium alloy induced by superlattice coating. Int. J. Fatigue.

[117] Bai Y., Guo T., Wang J., Gao J., Gao K., Pang X. (2021). Stress-sensitive fatigue crack initiation mechanisms of coated titanium alloy. Acta Mater..

[118] Zhang Z., Zhang Y., Zhang Z., He G. (2023). Effect of brittle TiN coating on fatigue performance of TC11 titanium alloy under rotating bending and tension-tension. J. Alloys Compd..

[119] Zhang Z., Yang M., He G. (2024). Effect of TiN monolithic and Ti/TiN multilayer coating on the fatigue behavior of titanium alloy under tension-tension. J. Mater. Res. Technol..

[120] Apachitei I., Lonyuk B., Fratila-Apachitei L.E., Zhou J., Duszczyk J. (2009). Fatigue response of porous coated titanium biomedical alloys. Scr. Mater..

[121] Bortolan C.C., Campanelli L.C., Bolfarini C., Oliveira N.T.C. (2016). Fatigue strength of Ti-6Al-4V alloy with surface modified by TiO2 nanotubes formation. Mater. Lett..

[122] Shi H., Liu D., Jia T., Zhang X., Zhao W. (2023). Effect of the ultrasonic surface rolling process and plasma electrolytic oxidation on the hot salt corrosion fatigue behavior of TC11 alloy. Int. J. Fatigue.

[123] Campanelli L.C., Duarte L.T., da Silva P.S.C.P., Bolfarini C. (2014). Fatigue behavior of modified surface of Ti–6Al–7Nb and CP-Ti by micro-arc oxidation. Mater. Des..

[124] Pang Z., Cui L., He W., Liang X., Cao Z., Zhao W., Song J., Hu S., Luo S. (2024). Superior high-cycle fatigue property through in-situ precipitation of α’ martensite in additively repaired titanium alloy. J. Alloys Compd..

[125] Wang L., Luo S., Lu K., Zhang X., Zhao Z., Liu P., Yi M., Zhou L. (2024). Effect of laser additive repair on high cycle fatigue properties of TC17 titanium alloy. Int. J. Fatigue.

[126] Costa M.Y.P., Venditti M.L.R., Voorwald H.J.C., Cioffi M.O.H., Cruz T.G. (2009). Effect of WC–10%Co–4%Cr coating on the Ti–6Al–4V alloy fatigue strength. Mater. Sci. Eng. A.

[127] Lynn A., DuQuesnay D. (2002). Hydroxyapatite-coated Ti–6Al–4V: Part 1: The effect of coating thickness on mechanical fatigue behaviour. Biomaterials.

[128] Wang X., Zhang H., Liu B., Man T., Cui X., Liu T., Zhao T., Luhovskyi Y., Lu S., Nong Z. (2025). Design, preparation, and tribological properties of novel wide temperature range self-lubricating coatings on TC4 alloy. Surf. Coat. Technol..

